# Welcome note Republic of San Marino

**DOI:** 10.5334/ijic.1042

**Published:** 2012-09-04

**Authors:** Claudio Podeschi

**Affiliations:** Minister of Health San Marino

It is a pleasure to welcome and host you here in the Republic of San Marino.

In the 18th century, a Father of the Nation left us this motto: “known to us, unknown to the others”.

Probably this is not a good sentence to integration!

In the 20th century, everybody knows everybody: you can discover an organization by clicking a web site.

In the past, we survived thanks to darkness, now we could disappear and die if nobody knew our existence and attributed our value to us.

So, this international meeting becomes part of a new way to understand welfare and integrated health care.

Knowledge and integration are now our means to introduce ourselves to the world.

We bet on our health care system as a passport to integration and to a new social and economic course for our Country.

We are aware that we can accept this challenge on the future by meeting our citizens' needs through an integrated health care system as a guarantee of quality and value.

Thank you and I wish you all a fruitful work.

